# Genomic transcriptional response to loss of chromosomal supercoiling in *Escherichia coli*

**DOI:** 10.1186/gb-2004-5-11-r87

**Published:** 2004-11-01

**Authors:** Brian J Peter, Javier Arsuaga, Adam M Breier, Arkady B Khodursky, Patrick O Brown, Nicholas R Cozzarelli

**Affiliations:** 1 Department of Molecular and Cell Biology, University of California, Berkeley, CA 94720-3204, USA; 2 Mathematics Department, University of California, Berkeley, CA 94720, USA; 3 Graduate Group in Biophysics, University of California, Berkeley, CA 94720, USA; 4 Department of Biochemistry, Molecular Biology, and Biophysics, University of Minnesota, St. Paul, MN 55108, USA; 5 Department of Biochemistry and Howard Hughes Medical Institute, Stanford University, Stanford, CA 94305-5307, USA; 6 Current address: Neurobiology Division, MRC Laboratory of Molecular Biology, Cambridge CB2 2QH, UK

## Abstract

**Background:**

The chromosome of *Escherichia coli *is maintained in a negatively supercoiled state, and supercoiling levels are affected by growth phase and a variety of environmental stimuli. In turn, supercoiling influences local DNA structure and can affect gene expression. We used microarrays representing nearly the entire genome of *Escherichia coli *MG1655 to examine the dynamics of chromosome structure.

**Results:**

We measured the transcriptional response to a loss of supercoiling caused either by genetic impairment of a topoisomerase or addition of specific topoisomerase inhibitors during log-phase growth and identified genes whose changes are statistically significant. Transcription of 7% of the genome (306 genes) was rapidly and reproducibly affected by changes in the level of supercoiling; the expression of 106 genes increased upon chromosome relaxation and the expression of 200 decreased. These changes are most likely to be direct effects, as the kinetics of their induction or repression closely follow the kinetics of DNA relaxation in the cells. Unexpectedly, the genes induced by relaxation have a significantly enriched AT content in both upstream and coding regions.

**Conclusions:**

The 306 supercoiling-sensitive genes are functionally diverse and widely dispersed throughout the chromosome. We propose that supercoiling acts as a second messenger that transmits information about the environment to many regulatory networks in the cell.

## Abstract 2

Microarray analysis shows that transcription of 306 *E. Coli* genes is affected by changes in the level of chromosome supercoiling, suggesting that supercoiling transmits regulatory signals from the environment to many cellular pathways.

## Background

The chromosome of *Escherichia coli *is a circular double-stranded DNA molecule that is maintained in a negatively supercoiled state. Supercoiling induces torsional tension in the DNA, and thus can influence processes that involve the opening of the double helix, such as replication initiation [1], DNA looping [2] and transcription [3]. A number of external stimuli, such as osmotic stress, oxygen tension, nutritional shifts, and temperature change affect supercoiling (for review see [4]), suggesting that supercoiling is a mechanism by which environmental changes could be communicated to the transcriptional machinery.

In *E. coli*, supercoiling is maintained at a precise range during log phase growth by the topoisomerases DNA gyrase, topoisomerase I (topo I), and topoisomerase IV (topo IV) [5-7]. DNA gyrase and topo IV are ATP-dependent type II topoisomerases that introduce negative supercoils and remove positive supercoils, respectively [8-10], whereas topo I is a type IA topoisomerase that removes negative supercoils [11]. Together, these activities remove the topological effects of translocating proteins, such as RNA polymerase, that create (+) supercoils in front and (-) supercoils behind the moving protein [12,13]. The balanced activities of these enzymes result in a steady-state level of negative supercoiling. In turn, supercoiling modulates the expression of the genes for gyrase (*gyrA *and *gyrB*), and for topo I (*topA*). Relaxation of the chromosome upregulates *gyrA *and *gyrB *and downregulates *topA *as a form of feedback control [14-16]. This dual response also indicates that (-) supercoiling can promote, as well as inhibit, gene expression. It is perhaps not surprising that transcription of topoisomerase genes may be sensitive to supercoiling changes. Yet transcription of other genes, such as *fis *(a nucleoid-associated protein and transcriptional regulator), *ilvG *(an amino-acid synthase subunit) and *cydAB *(an oxidase involved in aerobic respiration), has been found to be sensitive to supercoiling [17-19], suggesting that a wider class of genes whose expression is sensitive to supercoiling may exist. Furthermore, a recent search for osmotic shock genes found a cluster of genes with enhanced sensitivity to supercoiling [20]. If supercoiling is used as a mechanism to sense environmental changes, we predict that genes from many functional classes would be affected by supercoiling, because environmental changes such as temperature and osmotic strength will affect many different reactions in the cell. Determining which genes are supercoiling sensitive may illuminate principles of promoter activation, such as common sequence characteristics in promoters and regulation of transcription initiation [14,17,18].

In this study, we used cDNA microarrays [21,22] representing nearly the entire *E. coli *K-12 genome to systematically identify those genes that respond to relaxation of the chromosome during log-phase growth. We used antibiotics and mutations in the topoisomerase genes to change supercoiling levels by independent mechanisms and thus discerned the general effects of chromosome relaxation. We classify supercoiling-sensitive genes, or SSGs, according to their response to DNA relaxation. Therefore, we call 'relaxation-induced genes' those genes whose expression is increased upon DNA relaxation, and 'relaxation-repressed genes' those whose expression is repressed by DNA relaxation.

An extensive statistical analysis of our experimental results revealed 200 relaxation-repressed genes and 106 relaxation-induced genes; in total, around 7% of all genes in the genome were found to be significantly affected by supercoiling changes. Many of these genes are more sensitive to supercoiling than *gyrA *or *topA*, and their expression patterns correlated with the supercoiling level of a reporter plasmid in the cells. SSG transcripts have the same rates of RNA decay as non-SSG transcripts, and thus the changes in expression were due to a change in the rate of RNA synthesis, rather than RNA decay.

We discovered that the sequences of the relaxation-induced genes are significantly (*p *< 0.0001) AT-rich in their upstream sequences, and also have AT-rich coding regions. Relaxation-repressed genes had a corresponding preference for GC-rich sequences. The SSGs are dispersed throughout the chromosome, and fall into many different functional classes. We propose that the large number and functional diversity of the SSGs reflects the role of supercoiling as a second messenger that responds to environmental changes and feeds into different regulatory networks.

## Results

### Topoisomerase inhibition

We sought to determine the genes that are activated or repressed by relaxation of the (-) supercoils in the chromosome. To isolate the expression changes due to the loss of supercoiling from those due to the experimental approach, we used three different methods to relax the chromosome. In two of the methods we inhibited gyrase and topoIV with either quinolone or coumarin antibiotics, and in the third we used a temperature-sensitive strain in which gyrase is inhibited at 42°C. Because it is technically difficult to quantify the supercoiling state of the bacterial chromosome, we used a plasmid, pBR322, in the strains as a reference. Co-transcriptional translation of the *tetA *gene in pBR322 anchors this plasmid to the membrane [23], and thus this plasmid has been used as a model for the chromosome [7]. The superhelical density, or σ, of plasmids can be readily measured. Plasmid σ values for all of the relaxation experiments are shown in Table 1.

#### Inhibition of topoisomerases by norfloxacin

The quinolone antibiotic norfloxacin selectively and immediately inhibits gyrase and topo IV [24-26]. We used isogenic strains with resistance mutations in the genes for gyrase (*gyrA *and *gyrB*) or topo IV (*parC *and *parE*) as controls, to separate expression changes due to undiscovered drug targets from those directly due to changes in supercoiling. When we inhibited gyrase by treating *gyrA*^+ ^*parC*^*R *^cells with 15 μg/ml norfloxacin, the reporter plasmid in the cells was rapidly relaxed (Table 1). In a parallel experiment, plasmid DNA in a drug resistant *gyrA*^*R *^*parC*^*R *^strain remained (-) supercoiled. After 30 minutes, there was a 10^3^-fold drop in viability in the sensitive strain, but only a 17% drop in the resistant strain. A norfloxacin concentration of 50 μg/ml fully inhibited both gyrase and topoisomerase IV in the wild-type strain (data not shown), while the resistant strain retained wild type levels of (-) supercoiling and showed only a slight drop (15%) in viability, indicating that we did not overcome the drug resistance. At bacteriocidal concentrations similar to these, quinolones cause a decrease in the sedimentation coefficient of the nucleoid, indicating relaxation of the chromosomal supercoils [27]. The reference RNA sample was from cells removed immediately before addition of the drug (*t *= 0) and was labeled with Cy3 (green). RNA samples taken 2, 5, 10, 20 and 30 minutes after drug addition were labeled with Cy5 (red). The labeled experimental and reference samples were mixed in equal amounts before hybridization to a microarray.

Inhibition of topoisomerases by quinolones leads to double-strand breaks in the chromosome [28]. Thus, norfloxacin not only reduces supercoiling, but also induces the SOS response to DNA damage [29]. We found that the induction of the SOS response by norfloxacin was significantly slower and less extensive than either the responses of the SSGs (see below) or the SOS induction caused by UV treatment (see Additional data file 1). We conclude that the induction of SOS by norfloxacin is not a significant impediment to our search for SSGs.

#### Inhibition of topoisomerases by a coumarin antibiotic

We also relaxed the chromosome using novobiocin, a coumarin antibiotic that inhibits gyrase, and at a higher concentration, topo IV [30,31]. Novobiocin inhibits the ATPase activity of the enzyme [32,33], and the mechanism of inhibition is completely different from that of norfloxacin [34]. We treated cells with 20, 50 and 200 μg/ml novobiocin for 5 minutes and measured the DNA relaxation by gel electrophoresis (Table 1) and the gene-expression changes by microarray. In addition to changes due to a loss of topoisomerase activity, we saw changes in a set of non-overlapping genes between the norfloxacin and novobiocin experiments, indicating that there are also drug-specific transcriptional effects. Since we focused our analysis on the genes that responded to supercoiling independent of the relaxation method used, these drug-specific changes were removed from consideration.

#### Inhibition of gyrase by mutation

We also used a mutant that is temperature-sensitive for gyrase activity [35], which results in relaxation of the chromosome at the restrictive temperature [36]. We measured expression changes in *gyrB234 *cells upon shift to the restrictive temperature and subsequent relaxation of the DNA (Table 1). To control for the effects of the temperature shift on gene expression, we compared the changes in the *gyrB*^*TS *^mutant to those in an identically treated isogenic wild-type strain. The *gyrB*^*TS *^data were combined with the norfloxacin and novobiocin data to make a body of experiments and controls where the transcriptional effects of relaxation were isolated from effects due to the method used to relax the chromosome.

### Identification of supercoiling-sensitive genes by statistical analysis

We obtained a dataset from a total of 35 arrays. Fourteen of the arrays were controls in which either drug was added to resistant cells or the temperature was shifted for wild-type cells. The supercoiling of the reporter plasmid did not change in these controls (Table 1). The remaining 21 arrays represented experiments in which the DNA was relaxed by different methods and over various time courses. This rich dataset allowed us to use statistical methods to determine those genes whose expression significantly varied with supercoiling levels.

Using threshold ratio values (for example, requiring a twofold change in expression) to determine which genes change significantly during an experiment can bias expression analysis towards genes with very low or variable expression levels [37]. We used statistical methods to minimize the bias. To assess the significance of the difference in gene expression between supercoiled and relaxed samples we used the method described by Dudoit *et al*. [38]. Briefly, we performed a *t*-test for each gene and corrected the obtained *p*-values for multiple testing by a step-down procedure [39]. The corrected *p*-value represents the probability that the differences in gene expression between the controls and relaxation experiments could have arisen by chance, after taking the expression of all genes into consideration. We obtained *p*-values ranging from 0.000125 to 1.

As an independent metric of supercoiling sensitivity, we measured how closely gene expression followed the level of DNA supercoiling, by calculating the correlation of the expression of each gene across all of the experiments with the level of supercoiling in the reporter plasmid. Relaxation-induced genes showed a positive correlation with plasmid linking number (that is, as (-) supercoiling is lost, both linking number and gene expression increase), up to a maximum observed Pearson correlation coefficient of 0.91. Relaxation-repressed genes showed a negative correlation with plasmid linking number to a minimum Pearson coefficient of -0.88. The majority of genes (3,190, or 80%) showed no strong correlation with plasmid supercoiling, resulting in Pearson coefficients between 0.5 and -0.5. The *p*-value represents the robustness of the response to relaxation, whereas correlation with plasmid supercoiling may represent sensitivity to changes in supercoiling levels. For example, a gene that is always completely repressed in response to relaxation will have a low *p*-value, but may show little correlation with intermediate levels of supercoiling. Similarly, a gene with more variable expression may have a higher *p*-value, but may also have a higher sensitivity to intermediate supercoiling levels. Taken together, these metrics provide a detailed account of supercoiling sensitivity.

**Figure 1:**
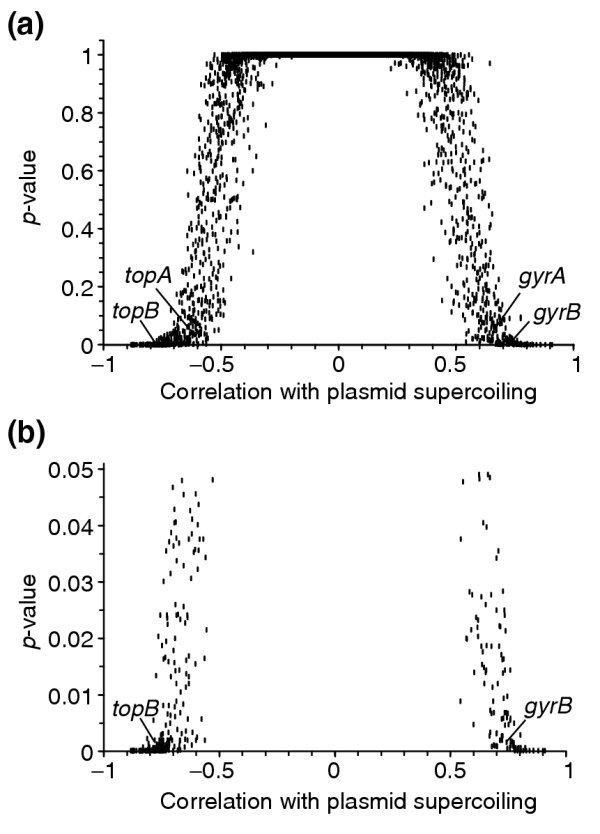
Significance versus correlation of gene expression and plasmid supercoiling values for all genes over all experiments. For each gene we computed the correlation coefficient between its gene expression ratios (base 2 logarithm) over all experiments with the superhelical density (σ) of a reporter plasmid, as measured by gel electrophoresis. These values are plotted against the *p*-value, which represents the chance that the difference in expression between relaxation and control experiments could have arisen randomly. **(a) **Scatter plot for all genes. There is a general trend in which genes with low *p*-values showed very high correlation (absolute value) between expression and plasmid supercoiling. The points corresponding to the topoisomerase genes *gyrA*, *gyrB*, *topA *and *topB *are indicated. **(b) **Expanded portion of (a) highlighting those genes classified as significant (*p *< 0.05). Genes with very low *p*-values show high positive or negative correlation between expression and plasmid supercoiling.

The *p*-values for all of the genes versus their correlation to plasmid supercoiling are plotted in Figure 1a. The great majority of the genes have both high *p*-values and little correlation with plasmid supercoiling. Those genes with the lowest *p*-values (and thus, the most significant expression change upon relaxation) tended to be more strongly correlated (or anticorrelated) to plasmid supercoiling. The data for all genes can be found in Additional data file 2. Among all genes there is a continuous variation in both *p*-value and correlation to plasmid supercoiling. We found a total of 306 genes at *p *< 0.05, which we define as SSGs. Of these, 106 genes were induced by DNA relaxation and have a positive correlation with plasmid linking number, while 200 genes were repressed by relaxation and these have a negative correlation with plasmid linking number. The correlations of the SSGs with plasmid supercoiling are shown in Figure 1b, which is an expansion of the significant region of the plot in Figure 1a. All the SSGs have a correlation with plasmid supercoiling with an absolute value greater than 0.5, which validated our selection on the basis of *p*-value. Just over half of the SSGs have high significance, *p *< 0.005. The high redundancy of our data (21 arrays measuring responses to DNA relaxation, and 14 control arrays with negatively supercoiled DNA) minimized the influence of any single array measurement. Thus we can be confident that the genes we classed as SSGs have a reproducible response to supercoiling changes.

**Figure 2:**
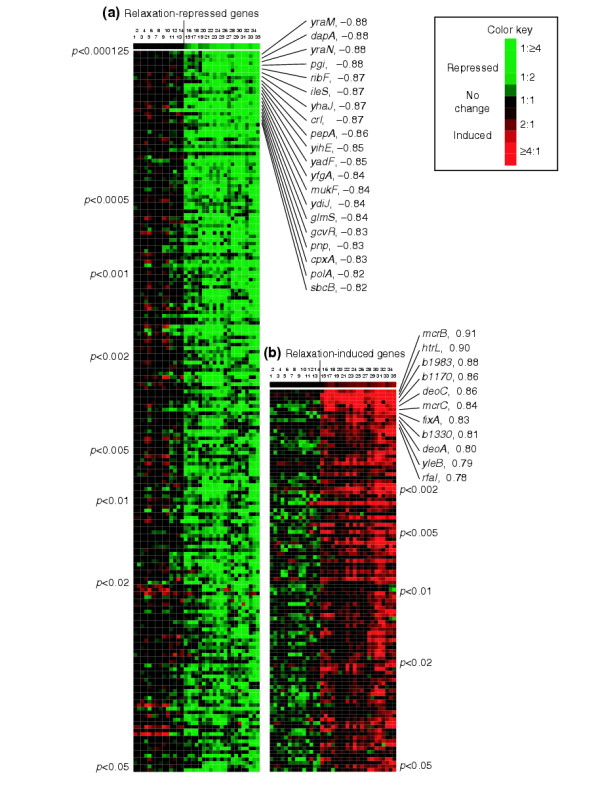
Expression profiles of relaxation-induced and repressed genes. The figure shows a cluster diagram ordered according to the *p*-value of each gene (from 0.000125 to 0.05). Each row represents a gene and each column an experiment. Therefore, each of the entries of the array shows the expression level for a gene in a given experiment. **(a) **Relaxation-repressed genes; **(b) **relaxation-induced genes. The set of experiments labeled 1 to 14, to the left of the vertical mark in (a and b), represents the control set in which plasmid supercoiling did not change. Experiments to the right of the vertical mark, labeled from 15 to 35, are experiments in which the chromosome is relaxed. As experiments were done in a time-dependent fashion, red color means that gene expression is higher at time points after relaxation of the chromosomes, while green means the opposite. Black indicates no change in expression during the experiment. Columns 1-5, gene expression measured after addition of 15 μg/ml norfloxacin to a norfloxacin-resistant strain at times *t *= 2, 5, 10, 20 or 30 min; columns 19-27, gene expression measured after addition of 15 μg/ml norfloxacin to an isogenic wild-type strain at times *t *= 2, 3, 4, 5, 7, 10, 15, 20 or 30 min; columns 6-10, gene expression at times *t *= 2, 5, 10, 20 or 30 min after addition of 50 μg/ml norfloxacin to a norfloxacin-resistant strain; columns 28-32, gene expression at these times after addition of the same concentration of norfloxacin to an isogenic wild-type strain; columns 15-18, gene expression at times *t *= 2, 5, 10 or 20 min after temperature shift in a temperature-sensitive mutant strain; columns 11-14, gene expression at times *t *= 2, 5, 10 or 20 min after temperature shift in an isogenic wild-type strain; columns 33-35, gene expression at fixed time *t *= 5 min and varying concentrations of novobiocin (Novo) = 20, 50 or 200 μg/ml on a wild-type strain. A total of 200 genes are repressed in response to DNA relaxation, while 106 genes are induced. The top row is a model expression profile of the supercoiling of the reporter plasmid in each experiment (Table 1). *p*-values and correlation coefficients with plasmid supercoiling levels for the top 10% of genes in each class are listed. The complete expression data for each gene can be found in Additional data file 2.

Figure 2a shows the expression changes in the 200 relaxation-repressed genes across the 35 conditions tested, with each numbered column representing one array. Each row represents the expression of one gene across all experiments, ranked by *p*-value (from the top). Each colored entry in the diagram corresponds to one spot on one array (that is, expression of a gene for a point in a given experiment: red if expression increased during the experiment, green if it decreased). Conversely, these relaxation-repressed genes should have low ratios (and black squares) in the control experiments 1 to 14. The significant difference in SSG expression between the controls and relaxation experiments is reflected by the sharp contrast between the mostly black controls and the bright green relaxation experiments. At the top we have shown a model expression profile representing the supercoiling of the reporter plasmid in each experiment (Table 1), with black indicating no change in plasmid supercoiling and bright green indicating complete relaxation of the plasmid. These plasmid relaxation data match very well the expression data of the SSGs. The names of the top 10% of genes (those with the lowest *p*-value) are listed, along with their correlations to plasmid supercoiling levels.

The 106 genes that are induced by relaxation are similarly shown in Figure 2b. Red squares indicate expression at a higher level when the DNA is relaxed. Once again there is a striking difference in color between the control and relaxation experiments, and the SSGs show a strong similarity to the model profile at the top (in this model profile, red color indicates relaxation of the reporter plasmid). Several of the relaxation-induced genes are marginally repressed (shown by green color) in some control experiments. This is due to the fact that our statistical selection did not require the SSGs to be unchanged in the controls, but only required a significant difference in expression between the controls and relaxation experiments. However, this trend highlights the large expression change (from repression to induction) caused by chromosomal relaxation. It is striking how many genes respond significantly to a loss of chromosomal supercoiling (7% of the total genes). The full list of SSGs, with their *p*-values, correlations to supercoiling, and expression levels in each experiment can be found in Additional data file 3.

### Kinetic analysis of gene expression and supercoiling

**Figure 3:**
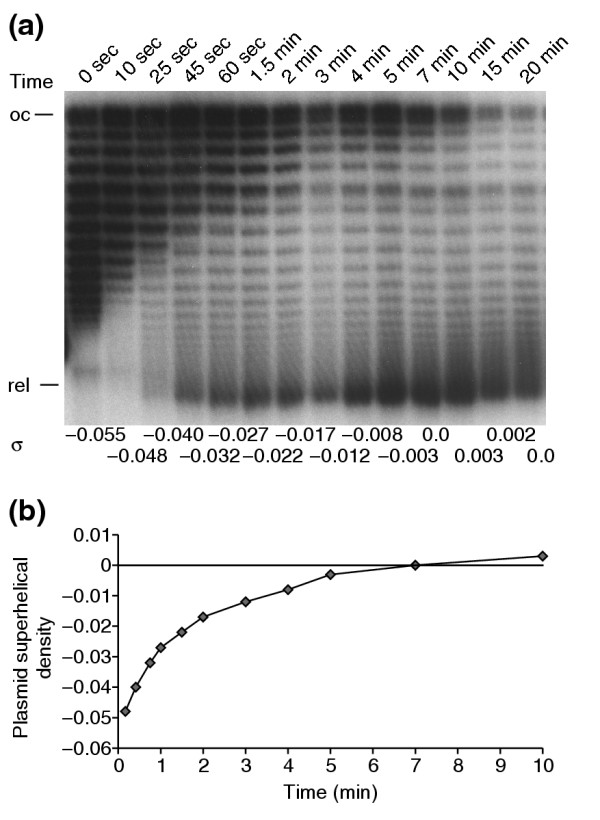
Plasmid relaxation kinetics. *gyrA*^+^*parC*^+ ^cells were treated with 15 μg/ml norfloxacin for the indicated times before samples were removed for DNA and microarray analysis. **(a) **pBR322 plasmid DNA was isolated and run on a 1% agarose gel + 2.8 μg/ml chloroquine to provide an indicator of topoisomerase activity in the cells. The positions of open circular (oc) and relaxed (rel) marker plasmids on the gel are shown. The distribution of native (-) supercoiled DNA is shown in lane 1. As the plasmid becomes relaxed, the center of the distribution first moves toward the open circular form and then moves down the gel to the relaxed position. The calculated superhelical density values for the plasmids (σ) are shown at bottom of each lane. **(b) **Graph of the average σ values from (a). Values of σ stabilized around 0 for times greater than 10 min and are not shown.

**Figure 4:**
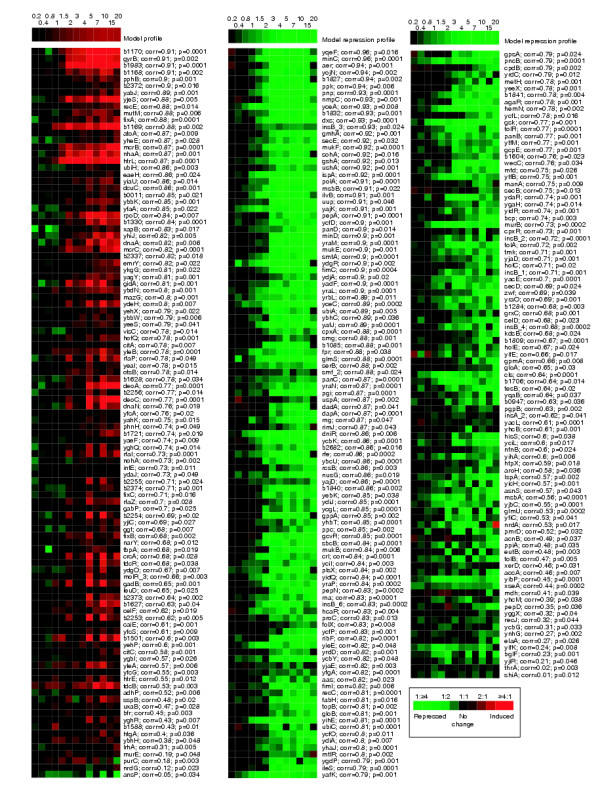
Kinetics of the expression changes of the supercoiling-sensitive genes. Norfloxacin was added to wild-type *E. coli *cells and RNA was extracted from cells removed from the culture at the time points shown (in minutes) above each column. This diagram illustrates the kinetics of the SSG responses, which are ranked by their correlation to plasmid supercoiling levels in this experiment (see Figure 3). *p*-values and correlation coefficients for each gene are listed (see Materials and methods for calculation). The model profiles shown at the top are colored representations of plasmid supercoiling levels, as in Figure 2. Red squares indicate that a gene is induced during the experiment, green squares that a gene is repressed.

We expected that changes in SSG expression that are a direct effect of supercoiling changes (rather than mediated through other genes) should respond quickly to relaxation. We used a finer time-course experiment to determine which genes had the fastest response to chromosomal relaxation. When 15 μg/ml norfloxacin was added to *gyrA*^+^*parC*^+ ^cells, plasmid supercoiling levels changed dramatically within the first minute (Figure 3). Significant changes in gene expression followed by 2 minutes (Figure 4). We ranked the SSGs according to their correlation to plasmid supercoiling levels in this experiment. Thus, genes with transcriptional changes that match the kinetics of plasmid relaxation have high correlations. About 90% of the SSGs had a correlation higher in absolute value than 0.5, and more than half had correlations better than 0.75. The expression profiles of all of the SSGs, ranked by their correlation to plasmid supercoiling, are shown in Figure 4. The correlation of the SSGs to plasmid relaxation kinetics shows the sensitivity of gene expression to changes in supercoiling, while the *p*-value is a good indicator of the reproducibility of the response to supercoiling across the different experimental conditions we tested.

The speed of the transcriptional response to relaxation, combined with the strong correlations to supercoiling of the reporter plasmid in the cells, is strong evidence that the SSGs are directly regulated by supercoiling changes. Furthermore, given that *E. coli *mRNAs have a mean half-life of 5.2 ± 0.3 minutes in LB media [40], RNA synthesis of the relaxation-repressed genes must have slowed almost immediately upon DNA relaxation, in order to produce the quick changes we recorded (Figure 4). More than half of the relaxation-repressed genes changed by twofold or more in the first 5 minutes of this experiment.

We found no correlation of *p*-value with the published values of RNA half-life [40] and in general the mRNA half-lives of the SSGs were not significantly different from those from the rest of the genome (data not shown). We conclude that the changes in SSG expression are direct effects on transcription, rather than an effect on RNA degradation.

### Sequences surrounding the start codon of supercoiling sensitive genes

**Figure 5:**
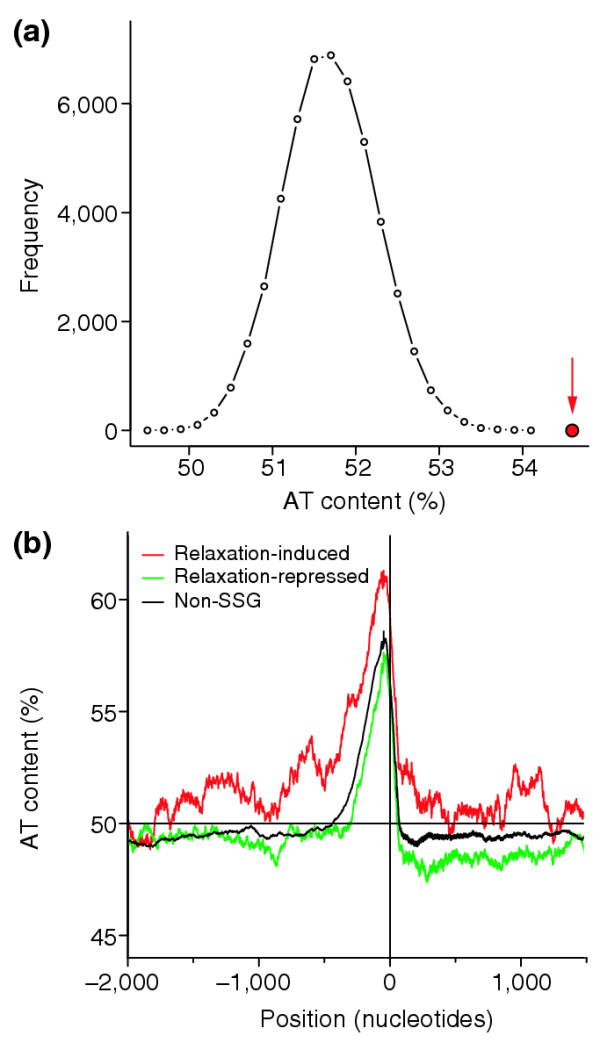
Analysis of AT content in upstream regions of SSGs. **(a) **The average upstream AT content of 50,000 groups of 106 randomly selected genes. The actual average upstream AT content of the group of 106 relaxation-induced genes (red circle) lies well outside the distribution. **(b) **Average AT content in a 100-nucleotide window is plotted against distance from the start codon for relaxation-induced (red), relaxation-repressed (green) and all other (black) genes for 300 nucleotides to either side of the translation start site. The *y*-axis is drawn at the first nucleotide of the start codon, and a horizontal line indicates 50% AT content. The relaxation-induced genes show a significantly increased AT content relative to the other sets of genes both before and after the start codon. The relaxation-repressed genes show a milder depression of AT content over this region, which is still significantly different from the rest of the genome. We found no significant differences outside the region shown.

We searched for a basis of supercoiling sensitivity at the nucleotide sequence level by examining the AT content in and around the SSGs. We considered only the first genes in an operon. Whereas relaxation-repressed genes have a slightly depleted AT content both upstream of their promoters and within the coding sequence, relaxation-induced genes have an emphatic elevation of AT content in similar regions. The AT content of relaxation-induced genes from 800 nucleotides before to 200 nucleotides after the start codon is 54.6%, compared with 51.7% for non-SSGs. To illustrate the very low probability of selecting by chance a set of genes with such an elevated AT content, we randomly selected groups of first-in-operon non-SSGs 50,000 times and calculated AT content within the same window. We never found a set with an AT content as high as the relaxation-induced genes (red circle, Figure 5a). The difference in AT content is highly statistically significant (*p *= 3E-4).

This is not the only region in which the AT content of SSGs deviates from the rest of the genome. Figure 5b shows the mean AT content in a 100-nucleotide window for relaxation-induced, relaxation-repressed, and non-SSGs from 2 kilobases (kb) upstream to 1.5 kb downstream of the start codon. Nearly all genes, including non-SSGs, have elevated AT content upstream and just downstream of the start codon. The relaxation-induced genes, however, have a higher maximum AT richness and the elevated AT content extends over a wider region. Also, the relaxation-repressed genes showed a highly statistically significant reduction in AT content from -400 to +1,000 relative to the start codon (*p *= 1E-6).

Striking as these differences in AT content are for SSGs as a group, they are not sufficient to distinguish an individual SSG from a non-SSG. That is, not all genes with high or low AT content were supercoiling sensitive in our experiments. Although such genes are rare in the non-SSG population, the greater size of the pool of non-SSGs results in many genes with wide variations in AT content. Also, supercoiling sensitivity cannot solely be due to differences in AT content, as a few SSGs were highly sensitive to supercoiling changes in spite of having an AT content similar to the rest of the genome.

## Discussion

In this analysis of supercoiling effects on transcription, we identified 306 genes that quickly and reproducibly respond to chromosomal relaxation. The comprehensive nature of our investigation, with responses of 93% of the genome (4,003 protein-coding genes) in 21 different relaxation experiments and 14 control experiments, allowed us to be more stringent than previous studies in our definition of SSGs, and to identify those genes that had statistically significant changes after the chromosome was relaxed by different methods. Genes that are sensitive to relaxation but are also affected by temperature shifts (including *topA *[41] and *gyrA *[42]) showed changes in our control experiments, and thus had less significant *p*-values. Accordingly, although the topoisomerase genes *topA *and *gyrA *both clearly respond to supercoiling (see Figure 1 and [14-16]), they have *p*-values of 0.058 and 0.062, respectively (compared to the *p*-value of 0.001625 for *gyrB*). The omission of these topoisomerase genes from our list of SSGs reflects the conservative statistical approach we used to define the list. There are probably other genes that respond to supercoiling changes in different conditions from those we tested (log-phase growth in rich media). Also, we defined SSGs by focusing on the immediate effects of relaxation, and thus considered only primary transcriptional changes, rather than downstream effects mediated by other gene products (though we note that 14 of the SSGs encode known transcriptional regulators). When downstream effects are considered, changes in supercoiling are likely to affect transcription of an even greater proportion of the genome.

There have been several previous attempts to measure the effects of supercoiling on gene expression in *E. coli*. Two early studies used either nylon membranes or two-dimensional protein gels to compare topoisomerase mutants with slightly different homeostatic levels of supercoiling, and neither study found a large number of genes [43,44]. This could be due to the lower sensitivity of these earlier studies and because they measured steady-state gene expression, generations after the initial mutations and subsequent adjustment to the new supercoiling levels.

A more recent analysis by Church and colleagues used microarrays to gauge the osmotic stress response of *E. coli *[20]. Surveying 2,146 genes that were above their threshold of detection, the authors scored a subset of 30 genes that should be significantly enriched for supercoiling-sensitive transcription. Four of the genes identified are on our list of SSGs (*ynhG*, *yrbL*, *otsB *and *yifE*). Seven others had *p *< 0.1 in our relaxation experiments, and the rest had still higher *p*-values in this study. It is possible that these genes are only responsive to supercoiling changes in the context of osmotic stress.

Just as supercoiling is affected by many environmental changes, such as osmotic shock, oxygen tension, nutrient upshift and temperature change, so too do changes in supercoiling affect genes in a large number of classes. Not surprisingly, a substantial fraction (6.9%) of the SSGs encode genes involved in DNA replication, recombination, modification and repair. However, the SSGs span many other classes, and thus are well positioned to feed into many different regulatory networks. Thus, supercoiling can act as a second messenger that quickly translates environmental changes to transcriptional programs, inducing and repressing specific genes independently of protein synthesis.

Several of the SSGs warrant further inspection. For example, the repression of the *smtAmukBEF *operon on loss of supercoiling is intriguing, given the importance of *mukB*, *mukE *and *mukF *in chromosome supercoiling and segregation [45,46]. Consistent with this, the XerC site-specific recombinase, which is needed for proper chromosome partitioning, is also repressed by relaxation. As (-) supercoiling promotes chromosome segregation in *E. coli *[47], these genes may represent part of a supercoiling 'checkpoint' that senses whether supercoiling levels are sufficient for proper chromosome segregation. Thus, if there is insufficient (-) supercoiling to support chromosome segregation, transcription of these genes may be suppressed until supercoiling is re-established.

Another relaxation-repressed gene that may be involved in chromosomal maintenance is *yrdD*, a 'putative topoisomerase'. *yrdD *encodes a 19 kilodalton (kDa) protein 30-40% identical to the carboxy-terminal domain of topoisomerase I from *Bacillus subtilis*, *Helicobacter pylori *and *Methanococcus jannaschii*. The function of YrdD is unknown, but the repression by chromosomal relaxation provides an intriguing lead.

Chromosomal relaxation leads to the repression of *cls *(cardiolipin synthase) and *ileS *(isoleucine tRNA synthetase), which is consistent with the earlier discovery that these genes were involved in sensitivity to gyrase inhibitors [48]. Also, we noted that the nucleotide salvage genes *deoA *and *deoC *are induced on relaxation. For these genes, DNA relaxation may be a signal of DNA damage, and their induction would allow the cell to recycle nucleotides necessary for DNA repair. Finally, the induction of *rpoD*, the σ^70 ^subunit of RNA polymerase, may help the cell compensate for the increased difficulty of melting the relaxed DNA template.

What is the basis of supercoiling sensitivity? Most of the well controlled analyses of supercoiling-sensitive promoters, notably of the *lacp*^*s *^and *ilv*_*G*_*P *[18,49-51], were done on plasmids *in vitro*. The more relevant issue is promoter regulation on chromosomes *in vivo*, where other factors may dominate. The CRP protein increases *lac *operon transcription at the low to moderate superhelicities found *in vivo*, and the nucleoid-associated protein IHF is implicated in the supercoiling sensitivity of the *ilvGMEDA *operon [52]. Also, the relative levels of the nucleoid-associated proteins IHF, H-NS and, especially, Fis, can influence the local topology of DNA and accordingly affect transcription of nearby promoters [53-55]. We found no significant enrichment of genes regulated by IHF, H-NS, or Fis in our list of SSGs. However, we found that chromosomal relaxation affected different promoters to varying extents, and it is possible that the effect of changes in supercoiling may be amplified or attenuated at specific promoters by the actions of DNA-binding proteins. Finally, the proximity of genes to surrounding promoters and other barriers to supercoil diffusion may affect the response to supercoiling. For example, the modulation of the *Salmonella leu-500 *promoter by supercoiling requires that the promoter is either on the chromosome or on a plasmid anchored to the cell membrane by transcription and translation of a gene such as *tetA *[23]. Further analysis of supercoiling-sensitive promoters will be more straightforward with the set of genes identified in this paper and our finding that relaxation-induced genes have an enriched AT content in the promoter and initially transcribed sequences.

It is striking that there are so many relaxation-induced genes that are relatively repressed when the chromosome is (-) supercoiled. This is surprising because (-) supercoiling should favor formation of an open promoter complex. The promoter regions of many of the genes induced by relaxation are AT rich, which will make it easier to form an open promoter complex even when the DNA is relaxed and the energy required is greater. Alternatively, the difference in AT content could reflect structural features such as curvature or flexibility. Curved sequences of DNA can influence the position of plectonemic supercoils, and thus could serve to localize a promoter sequence to the apex of a superhelical loop [56]. We note that the AT richness for the relaxation-induced genes extends on both sides of the transcriptional start site. It has been previously shown that promoter activity can be regulated by the initial transcribed sequence [57,58]. Moreover, in their analysis of the *gyrA *and *gyrB *promoters, Menzel and Gellert [14] found that base-pairs downstream of the transcriptional start were important for the supercoiling sensitivity of these promoters. These authors proposed that promoter clearance may be the rate-limiting step during relaxation-induced transcription of *gyrA *and *gyrB*. Promoter clearance has also been invoked in the mechanism of supercoiling sensitivity of some promoters *in vitro *[51]. As our group of relaxation-induced genes is AT rich over this region, we can extend this hypothesis to transcription of many relaxation-induced genes *in vivo*, and propose that promoter clearance is generally a key regulatory step for supercoiling sensitive transcription.

The AT-rich regions of our relaxation-induced genes extend downstream of the translational start site, and thus may involve transcription elongation in addition to promoter clearance. There is growing appreciation of the regulation of transcription elongation [59-61]. The AT-rich regions deep within the coding sequence of relaxation-induced genes may reflect such regulation; easily melted regions of DNA may facilitate the continued movement of RNA polymerase along a relaxed, covalently closed template. At a given level of (-) supercoiling, there is likely to be an optimum AT content that facilitates both unwinding and subsequent closure of the transcription bubble. This hypothesis is strengthened by the fact that the genes with the opposite response, the relaxation-repressed genes, have a significantly depressed AT content over the same region.

**Figure 6:**
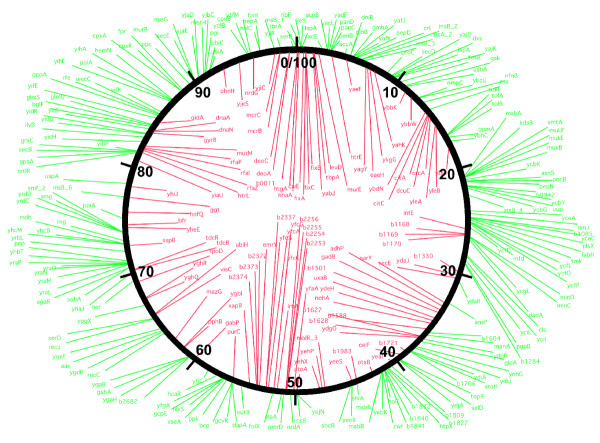
Chromosomal map of SSGs. Supercoiling-sensitive genes were mapped across the *E. coli *genome. Relaxation-induced genes are colored red and relaxation-repressed genes are in green. Genes are dispersed through the entire chromosome, making them good sensors for local changes of supercoiling of the chromosome.

The SSGs are useful as topological probes of the chromosome in living cells. While the SSGs are scattered throughout the chromosome, they are not evenly spread, but rather have regions of high and low density. The SSGs are plotted on a chromosomal map in Figure 6. The density of SSGs as a percentage of all genes in a 20-kb region varies from 2% to more than 20%. The regions with high SSG density may reflect spatial covariations in transcription which were recently described in the *E. coli *chromosome [62]. The distribution of the SSGs may also be influenced by the organization of the chromosome into topologically separate domains of supercoiling. We have already used the SSGs as local reporters of supercoiling to test the domains hypothesis. In recent work, we monitored expression from the SSGs after cleaving the chromosome with a restriction enzyme, and found that the SSGs accurately reported the resulting relaxation of the chromosome [63]. Relaxation diminished rapidly with distance from a restriction site, indicating that there are about 450 topologically separate domains in the chromosome. We also monitored transcription from the SSGs during replication in synchronized cells [64]. Here we found that the relaxation-induced and relaxation-repressed genes reported that supercoiling is re-established very quickly after the passage of the replication fork, again consistent with a large number of topological domains. Thus, the SSGs are not only a useful tool to study promoter regulation and the physiological effects of supercoiling changes, but also can lead to new findings about chromosome structure.

## Conclusions

We have shown that supercoiling acts as a transcription factor, with positive and negative effects on specific genes while leaving the majority of the genome unchanged. Like other transcription factors such as TrpR [65] and ArcA-P [66], supercoiling affects transcription from a wider array of genes than at first anticipated. The 306 genes that we identified as robust SSGs are classified into many different functional groups [67], including transcriptional regulators and genes in the SOS, PhoB and stringent-response regulons [68]. Transcriptional changes from the SSGs will affect a variety of transcriptional and regulatory networks, and thus supercoiling level is a global regulator that can affect a wide array of processes in the cell. As the topology of the chromosome is affected by anoxia, ionic strength and growth conditions, the cell can use supercoiling levels both to sense the environment and to effect appropriate transcriptional responses.

## Materials and methods

### PCR materials and conditions

Amino- and carboxy-terminal primers for protein-coding open reading frames (ORFs) of *E. coli *K-12, strain MG1655 (Sigma-Genosys), were generously supplied by Fred Blattner (University of Wisconsin) and Carol Gross (University of California San Francisco). ORFs were amplified from MG1655 genomic DNA using ExTaq polymerase (PanVera) and failed PCR reactions were attempted again using Platinum Taq (Invitrogen) or previous successful reactions as the DNA template. Ninety-six percent of the ORFs were successfully amplified. PCR conditions were set according to those supplied with the primers. DNA was precipitated with isopropanol and prepared for microarray printing as described in [69]. We did not include the RNA-coding genes on the arrays because primers for these genes were not initially available, though we note that some genes, such as *tyrT*, have been shown to respond to changes in supercoiling [70].

### Microarray printing and processing

Detailed instructions on slide preparation, microarray printing and processing microarrays can be found online [69]. 384-well plates were dried down between prints and resuspended in deionized water each time after the first print.

### RNA preparation and microarray hybridization

*E. coli *cells were grown with shaking in LB media to an OD_600 _= 0.45-0.55 at 37°C, or at 30°C for temperature-sensitive strains. Samples of cells were withdrawn at intervals and added to a 1/10 volume of either 95% ethanol plus 5% phenol or 2 M NaN_3 _to stop transcription. Cells were then quickly harvested by centrifugation in a microcentrifuge. The supernatant was aspirated and the pellets frozen in liquid N_2_. Total RNA was prepared using the Qiagen RNeasy mini kit, except that 4 mg/ml lysozyme and a 30 sec incubation was used in the first step. For each microarray, 20 μg total RNA was primed with 1-2 μg of random hexamers and labeled by reverse transcription in the presence of Cy3- and Cy5-conjugated dUTP (Amersham Biosciences). For each experiment or condition, a Cy5-labeled experimental sample was combined with a Cy3-labeled reference sample and hybridized to a processed microarray as described [69]. After 5-7 h hybridization, microarrays were washed and scanned at 10 μm resolution with a GenePix 4000A scanner (Axon Instruments).

### Image processing

Scanned array images were visually inspected, and non-uniform spots were excluded from further analysis. The background was subtracted from the images that were then (median) normalized using the Scanalyze 2.0 program, v. 1.44 (Michael Eisen, Lawrence Berkeley National Laboratory) such that the total fluorescence in each channel was equal.

### Data analysis

We tested several methods of imputation to estimate the values of spots missing due to hybridization defects (described in [71]), and after error analysis of the different methods we chose the weighted mean of K-nearest neighbors for K = 20. With this method we obtained a total of 4,003 genes, or 93% of the total number of *E. coli *genes, that could be considered for further study. Because we were interested in changes in expression levels due to variations in supercoiling rather than to drug or genetic effects, we used a two-sample comparison approach (comparing the mean over all relaxation experiments with that of the control experiments) rather than a factorial analysis approach. We tested two commonly used methods to determine differentially expressed genes in the comparison of two samples. We found that the method of Dudoit *et al *[38], which controls the family-wise error (that is, the probability of finding at least one false positive) was slightly more stringent for our data than that developed by Tusher *et al *[37].

### Northern analysis

Samples were run on formaldehyde-MOPS 1% agarose gels and blotted onto a nylon membrane [72]. ^32^P-labeled DNA probes for *gyrB *and *asnB *(as a loading control) were synthesized from their respective PCR products, and radioactivity was quantified by a phosphorimager.

### Assays of DNA topology

Plasmid DNA was extracted from cells by the alkaline lysis method [72] or the Qiagen spin miniprep kit. The norfloxacin-resistant mutants and the *gyrB234 *mutant are in a C600 strain background, but all strains used have been described in greater detail elsewhere [26,35,73]. To increase the intracellular concentration of novobiocin, we used a *ΔacrA *strain that greatly slows drug efflux [74]. The superhelical density, σ, of pBR322 was determined by band counting [75] from the mean of the topoisomer distributions to a relaxed, covalently closed reference plasmid (σ = 0) which had been relaxed with calf thymus topoI. σ of pBR322 was calculated with the formula σ =Δ Lk/Lk_0_, where Lk_0 _for pBR322 = 4,361 bp/10.5 bp/turn = 415. Samples were run on parallel 20-cm gels containing 0, 2.8 or 10 μg/ml chloroquine for 18-26 h at 48-52 V with constant buffer recirculation, which allowed visualization of the entire distribution of topoisomers. Gels were southern blotted [72], and hybridized with a ^32^P-labeled probe made from random priming of pBR322. Radioactive blots were quantified using a phosphorimager.

### Microarray validation

We tested the validity of our microarrays in three ways. First, we compared gene expression ratios measured with microarrays to values obtained by northern hybridization. We measured induction ratios for *gyrB *by both methods 5 min after addition of the gyrase inhibitor novobiocin to *ΔacrA *cells at 5, 20, 50 and 200 μg/ml. The microarray ratios for these concentrations were 2.3, 4.8, 4.9 and 6.3, respectively, while the ratios from northern hybridizations were 2.8, 4.7, 4.7 and 5.1. Second, as an internal control we compared the transcription of genes in 153 known polycistronic operons. We found no operons with genes that changed expression more than 1.5-fold in opposite directions (data not shown). Third, we compared two identically grown cultures with the same microarray (see Additional data file 4). We used two strains that were isogenic, except that one had point mutations conferring norfloxacin resistance on gyrase and topo IV. The correlation coefficient of the gene-expression levels was 0.982, indicating the negligible variation between the cultures. In contrast, when we treated cells with the gyrase inhibitor norfloxacin (see Additional data file 4), the correlation coefficient with respect to the untreated cells was only 0.391 and hundreds of genes showed large differences in expression. We conclude that gene-expression changes resulting from slight genotypic changes or experimental repeats were negligible compared with the changes resulting from topoisomerase inhibition, and that the *E. coli *microarrays are a reliable method for quantifying these changes.

### Selection of supercoiling-sensitive genes

We limited the list of SSGs to those whose expression difference between treatments and controls was statistically significant (*p*-values < 0.05) over a total of 35 experiments, in which DNA gyrase was inhibited with novobiocin, norfloxacin or by a mutation that rendered gyrase temperature-sensitive. Next we determined the correlation of gene expression with the σ of a reference plasmid in the same cells. To calculate the correlation of gene expression to plasmid supercoiling, we created a model profile made up of the ratio of plasmid σ in each experiment to plasmid σ in the (supercoiled) reference for that experiment (Table 1). The maximum ratio was scaled to 2.5, representing a σ of 0 (complete relaxation) and the minimum ratio was scaled to 1, representing native supercoiling levels (-0.06). The model repression profile is simply the inverse of the model induction profile. Changes of the arbitrary scaling values did not alter the results. Correlation coefficients in Figure 4 were calculated with respect to those 13 arrays only.

## Additional data files

The following additional data files are available with the online version of this paper: Additional data file 1 contains data on the induction of the SOS response to DNA damage; Additional data file 2 contains gene-expression ratios for all genes across all experiments; Additional data file 3 contains gene-expression ratios for supercoiling-sensitive genes across all experiments; Additional data file 4 contains data on the reproducibility of microarray measurement of RNA levels.

## Supplementary Material

Additional data file 1 Data on the induction of the SOS response to DNA damage Click here for additional data file

Additional data file 2 Gene-expression ratios for all genes across all experiments Click here for additional data file

Additional data file 3 Gene-expression ratios for supercoiling-sensitive genes across all experiments Click here for additional data file

Additional data file 4 Data on the reproducibility of microarray measurement of RNA levels Click here for additional data file

## Figures and Tables

**Table 1:** Plasmid supercoiling measurements from relaxation experiments

Genotype	Experimental treatment	Plasmid σ	Model ratio
*gyrA*^*R*^*parC*^*R*^	**15 μg/ml Nor, 0 min**	-0.057	1.0
*gyrA*^*R*^*parC*^*R*^	15 μg/ml Nor, 2 min	-0.057	1.0
*gyrA*^*R*^*parC*^*R*^	15 μg/ml Nor, 5 min	-0.057	1.0
*gyrA*^*R*^*parC*^*R*^	15 μg/ml Nor, 10 min	-0.057	1.0
*gyrA*^*R*^*parC*^*R*^	15 μg/ml Nor, 20 min	-0.060	1.0
*gyrA*^*R*^*parC*^*R*^	15 μg/ml Nor, 30 min	-0.060	0.9
*gyrA*^*R*^*parC*^*R*^	**50 μg/ml Nor, 0 min**	-0.057	1.0
*gyrA*^*R*^*parC*^*R*^	50 μg/ml Nor, 2 min	-0.057	1.0
*gyrA*^*R*^*parC*^*R*^	50 μg/ml Nor, 5 min	-0.057	1.0
*gyrA*^*R*^*parC*^*R*^	50 μg/ml Nor, 10 min	-0.058	1.0
*gyrA*^*R*^*parC*^*R*^	50 μg/ml Nor, 20 min	-0.059	1.0
*gyrA*^*R*^*parC*^*R*^	50 μg/ml Nor, 30 min	-0.061	0.9
			
*gyrB*^+^	**37°C**	-0.059	1.0
*gyrB*^+^	42°C, 2 min	-0.059	1.0
*gyrB*^+^	42°C, 5 min	-0.059	1.0
*gyrB*^+^	42°C, 10 min	-0.059	1.0
*gyrB*^+^	42°C, 20 min	-0.061	1.0
			
*gyrB*^*TS*^	**37°C**	-0.044	1.0
*gyrB*^*TS*^	42°C, 2 min	-0.023	1.6
*gyrB*^*TS*^	42°C, 5 min	-0.016	1.8
*gyrB*^*TS*^	42°C, 10 min	ND	ND
*gyrB*^*TS*^	42°C, 20 min	0.000	2.5
			
*gyrA*^+^*parC*^*R*^	**15 μg/ml Nor, 0 min**	-0.057	1.0
*gyrA*^+^*parC*^*R*^	15 μg/ml Nor, 2 min	-0.025	1.7
*gyrA*^+^*parC*^*R*^	15 μg/ml Nor, 5 min	-0.009	2.2
*gyrA*^+^*parC*^*R*^	15 μg/ml Nor, 10 min	-0.006	2.3
*gyrA*^+^*parC*^*R*^	15 μg/ml Nor, 20 min	-0.002	2.4
*gyrA*^+^*parC*^*R*^	15 μg/ml Nor, 30 min	0.000	2.5
			
*gyrA*^+^*parC*^+^	**50 μg/ml Nor, 0 min**	-0.057	1.0
*gyrA*^+^*parC*^+^	50 μg/ml Nor, 2 min	-0.016	1.9
*gyrA*^+^*parC*^+^	50 μg/ml Nor, 5 min	-0.002	2.4
*gyrA*^+^*parC*^+^	50 μg/ml Nor, 10 min	0.000	2.5
*gyrA*^+^*parC*^+^	50 μg/ml Nor, 20 min	0.007	2.8
*gyrA*^+^*parC*^+^	50 μg/ml Nor, 30 min	0.016	3.2
*gyrA*^+^*parC*^+^	**15 μg/ml Nor, 0 min**	-0.055	1.0
*gyrA*^+^*parC*^+^	15 μg/ml Nor, 10 sec	-0.048	1.1
*gyrA*^+^*parC*^+^	15 μg/ml Nor, 25 sec	-0.040	1.3
*gyrA*^+^*parC*^+^	15 μg/ml Nor, 45 sec	-0.032	1.5
*gyrA*^+^*parC*^+^	15 μg/ml Nor, 1 min	-0.027	1.6
*gyrA*^+^*parC*^+^	15 μg/ml Nor, 1.5 min	-0.022	1.7
*gyrA*^+^*parC*^+^	15 μg/ml Nor, 2 min	-0.017	1.9
*gyrA*^+^*parC*^+^	15 μg/ml Nor, 3 min	-0.012	2.1
*gyrA*^+^*parC*^+^	15 μg/ml Nor, 4 min	-0.008	2.2
*gyrA*^+^*parC*^+^	15 μg/ml Nor, 5 min	-0.003	2.4
*gyrA*^+^*parC*^+^	15 μg/ml Nor, 7 min	0.000	2.5
*gyrA*^+^*parC*^+^	15 μg/ml Nor, 10 min	0.003	2.6
*gyrA*^+^*parC*^+^	15 μg/ml Nor, 15 min	0.002	2.6
*gyrA*^+^*parC*^+^	15 μg/ml Nor, 20 min	0.000	2.5
			
*ΔacrA*	**0 μg/ml Novo, 0 min**	-0.057	1.0
*ΔacrA*	20 μg/ml Novo, 5 min	-0.011	2.1
*ΔacrA*	50 μg/ml Novo, 5 min	0.005	2.7
*ΔacrA*	200 μg/ml Novo, 5 min	0.011	3.0

pBR322 plasmid DNA was isolated from cells and analyzed by electrophoresis. Experimental treatments in bold indicate samples taken immediately before addition of drug or temperature shift, which were used as a reference for the following time points. Model ratios represent values derived from plasmid σ by taking the ratio of σ in each time point and dividing by σ in the reference, and scaling that value such that a sigma of 0 corresponds to a model ratio of 2.5. Nor, norfloxacin; Novo, novobiocin; SSG, supercoiling-sensitive gene; TS, temperature-sensitive. ND, not determined.
