# Supplementary material for: Genomic transcriptional response to loss of chromosomal supercoiling in Escherichia coli
Source: Genome Biol. 2004 Nov 1;5(11):R87. doi: 10.1186/gb-2004-5-11-r87 (PMC545778; doi:10.1186/gb-2004-5-11-r87)
Supplement: Additional data file 1 — Data on the induction of the SOS response to DNA damage [file gb-2004-5-11-r87-s1.pdf]

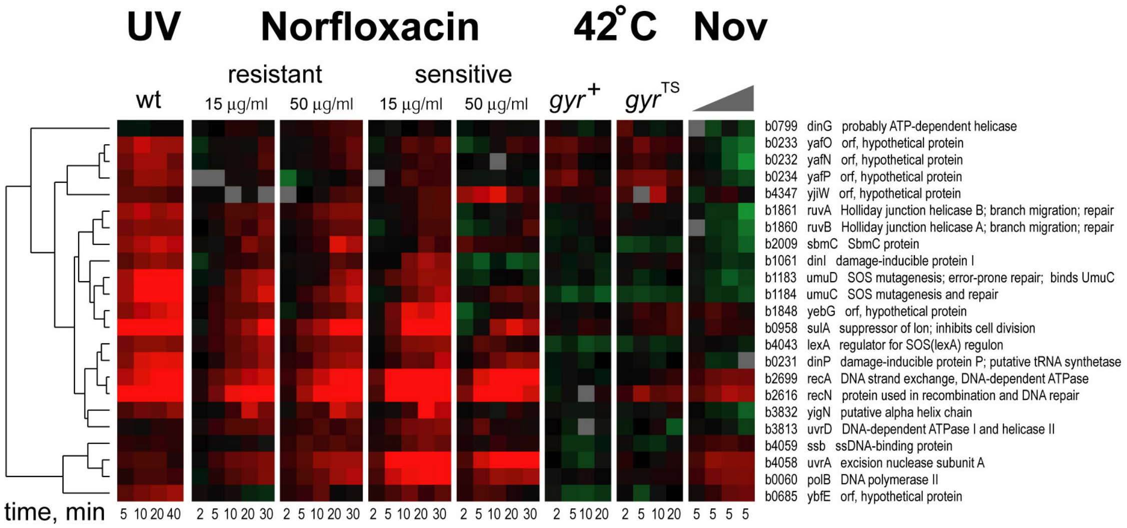

Additional data file 1. Induction of the SOS response to DNA damage.
The profile of SOS genes in DNA relaxation experiments is shown along with their expression after treatment of the cells with UV light [67]. A dendrogram representing the correlation coefficients of the gene expression profiles is shown at left; pairwise correlations within the node are greater than 0.8. Each row represents one gene, and each column is one timepoint. A red color indicates induction from the treatment, a green square indicates repression, and black squares correspond to no change. Genotypes are indicated at the top. Many of the known SOS genes were upregulated by norfloxacin addition, but this response was significantly delayed compared to the effects of UV treatment. Furthermore, the SOS response induced by norfloxacin was also delayed with respect to the changes we saw in the SSGs (Fig. 4). This is further evidence that the changes we saw in the SSGs were directly due to a loss of supercoiling, and not due to an effect of the norfloxacin treatment. Unexpectedly, many SOS genes induced in the wild type strain were also induced in a strain in which both gyrase and topo IV were mutated to norfloxacin resistance. This result is underlined by the viability assays and gel analysis of plasmid supercoiling, which indicated that norfloxacin resistance was not overcome at these concentrations. Thus, microarrays are a more sensitive indicator of the cellular response to topoisomerase poisoning. A second interesting finding is that novobiocin treatment induces a subset of SOS genes, even at 5µg/ml. Although novobiocin treatment does not cause double strand breaks in the DNA, the replication fork is markedly slowed by the inhibition of gyrase [9], and this seems sufficient to partially induce the SOS response.

Additional data file 1

BPeter et al.
