# Supplementary material for: Genomic transcriptional response to loss of chromosomal supercoiling in Escherichia coli
Source: Genome Biol. 2004 Nov 1;5(11):R87. doi: 10.1186/gb-2004-5-11-r87 (PMC545778; doi:10.1186/gb-2004-5-11-r87)
Supplement: Additional data file 4 — Data on the reproducibility of microarray measurement of RNA levels [file gb-2004-5-11-r87-s4.pdf]

Additional data file 4
B. Peter et al.

A.

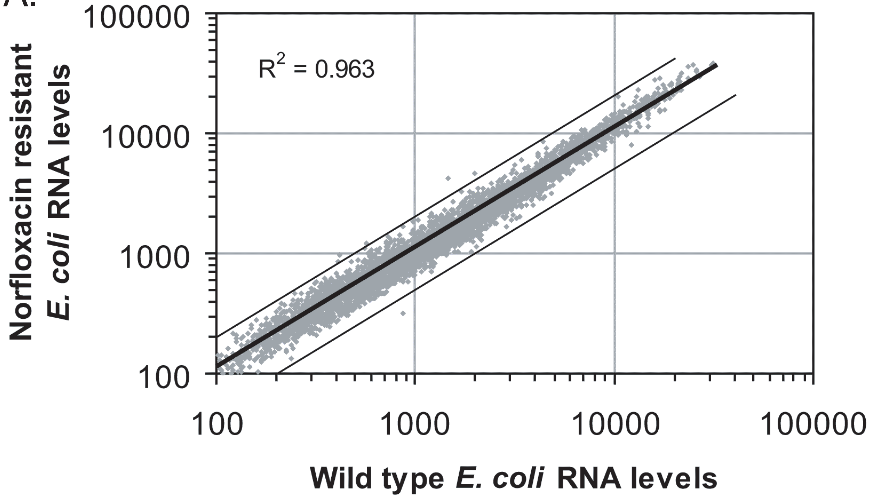

B.

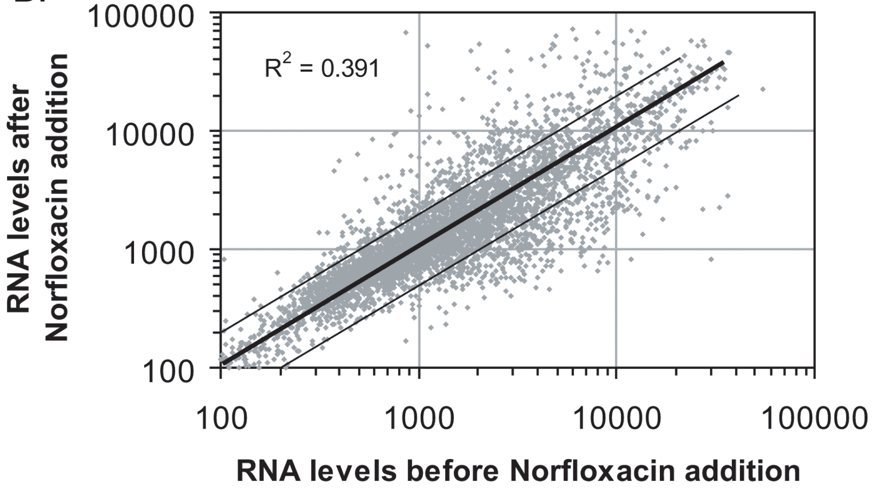

Additional data file 4. Reproducibility of microarray measurement of RNA levels.
A. Total RNA prepared on separate occasions from a norfloxacin resistant $gyrA^R parC^R$ strain and the isogenic wild type strain was labeled and applied to the same microarray. RNA from the resistant mutant was reverse transcribed into Cy5-labeled cDNA, and RNA from the wild type was reverse transcribed into Cy3-labeled cDNA. Fluorescence signals for all genes are plotted on a logarithmic scale (base 10), after background subtraction and removal of spots which had an extremely low signal in one or both channels. The thick line represents a linear best-fit line to the data, and hairlines represent twofold changes in either direction. The R^2 value is shown in the upper left. B. A similar quantification of a microarray experiment in which many genes changed their expression level. The wild type strain in A was treated with 15 microg/ml norfloxacin, and RNA from samples taken immediately before and 30min after the treatment was labeled by reverse transcription and applied to a microarray. The microarray data are plotted as in A. Points above the line represent genes that were induced by norfloxacin treatment, and points below the line represent genes that were repressed.
